# Bibliometric Analysis of Research on Telomere Length in Children: A Review of Scientific Literature

**DOI:** 10.3390/ijerph17124593

**Published:** 2020-06-26

**Authors:** Desirée Valera-Gran, Daniel Prieto-Botella, Paula Peral-Gómez, Miriam Hurtado-Pomares, Alicia Sánchez-Pérez, Eva-María Navarrete-Muñoz

**Affiliations:** 1 Department of Surgery and Pathology, Miguel Hernández University, 03550 Alicante, Spain; daniel.prieto@alu.umh.es (D.P.-B.); pperal@umh.es (P.P.-G.); mhurtado@umh.es (M.H.-P.); alicia.sanchez@umh.es (A.S.-P.); 2 Grupo de Investigación en Terapia Ocupacional (InTeO), Miguel Hernández University, 03550 Alicante, Spain

**Keywords:** telomere, childhood, scientometrics, scientific publication, genomic research, epidemiological research

## Abstract

Telomere length in early life has been recently associated with biological aging and development of negative consequences in later adult life. A relevant area of research has emerged to understand the factors that impact telomere length in children. We conducted a bibliometric analysis to track research output and identify global trends and gaps in the knowledge of telomere length in children. Bibliographic data were retrieved from the Web of Science database and then analyzed by using Bibliometrix R package. A total of 840 publications were yielded from 1991 to 2019. The references were prominently published in journals, with 20 high ranked journals contributing to 30% of literature on telomere length in children. The USA was the most productive country (35.7%), followed by Europe (12.1%), and Asia (11.9%). A knowledge map of telomere length in children through keyword analyses revealed that there were two potential main lines of research based on two different approaches: genomic research and epidemiological research. This study shows that telomere length in children is a topic of research that has gained significant relevance in the last decade. This bibliometric study may be helpful in identifying research trends and finding research hot spots and gaps in this research field.

## 1. Introduction

The telomere is a DNA-protein complex at chromosome ends that plays an essential role in maintaining and protecting chromosomal and genetic stability. The enzyme telomerase acts as a protective mechanism by adding telomeric repeat sequences to the ends of chromosomes, thereby preventing oxidative stress and cellular damage [1]. However, this protective physiological function does not remain constant. There are also damage causing processes that occur and can prompt the shortening of telomeres. In this regard, it is well known that DNA replication cycles naturally shorten telomeres over the human life span, therein telomere length is generally considered as a good biomarker of biological aging [2]. 

To date, a large number of studies have documented that most human aging-related diseases, such as poor immune function, cancers, cardiovascular disease, diabetes, mental/cognitive disorders, or depression, have been associated with short telomeres and telomere dysfunction [1]. At the same time, research focused on the mechanisms underlying telomere length has indicated that oxidative stress and inflammation are directly involved in telomere attrition. These biological processes usually precede the onset of chronic diseases [1,3], suggesting that a reduced telomere length may be a potential causal link connecting early adversity and later disease [4,5]. Excepting the well-established influence of age, a considerable number of genetic and non-genetic factors, mainly environmental factors, evaluated at different life stages have been determined as decisive predictors of telomere maintenance [4,6]. Importantly, telomere length in early life has been recently shown to be a significant determinant of biological aging as well as pivotal contributor to the development of negative consequences in later adult life [4,5]. In response to the traditional view of telomere length as a passive biomarker of human aging, new approaches based on a deep understanding of telomere biology in newborns and children have emerged in recent years [7]. These new approaches represent a significant shift in the understanding of telomeres from a static viewpoint, mainly focused on identifying problems of chromosome management, to a dynamic viewpoint that seeks to understand baseline sources of variation in telomere length [8]. From this biological perspective, the knowledge of telomere length in early life is considered crucial to elucidate the origins and diversity of telomere dynamics [9,10] and requires working within a transdisciplinary framework that includes a wide range of sciences [8,11]. In this regard, this period of life has been shown critically important in the field of telomere epidemiology for its decisive role in telomere attrition, especially, for its strong dependence on genetic and environmental factors [9,10,12]. There is a considerable number of studies focused on this life stage, which suggests that a growing body of research on the factors that impact on telomere length in children is becoming relevant to the development of this research field [12]. However, the evolution of generated knowledge in this research field still remains unexplored.

Bibliometric analysis in health sciences can be used to evaluate the development of publication in a specific area of research in order to identify global trends and gaps of knowledge. This type of analysis permits one to assess the impact and influence of scientific work by tracking citations [13]. On this occasion, citation analysis can specifically offer measures about the performance of research on telomere length in children to disclose trends in research output, countries of publication, and international collaborations. The quantitative evaluation of publication and citation data using bibliometrics can be useful to illustrate the trajectory of this specific field of research including which countries are producing research papers about telomere length in children and the study areas within research on telomere length during childhood, thereby providing an in-depth insight into the change in output and impact over time of this field of research. However, as far as we know, no bibliometric analysis of research on telomere length has been conducted so far. Consequently, the role of telomere length in children has not yet been systematically evaluated using this method of analysis.

The aim of this study was to present an overview of the research trends on telomere length in children up to now and elucidate the main points of future research directions. Using a bibliometric analysis, we examined the growth and citation of publications, active authors, countries and institutions, international collaboration, and the frequency of terms.

## 2. Materials and Methods

### 2.1. Search Strategy and Data Extraction 

In this study, the literature research for all the published articles on telomere length and children was performed using the Web of Science (WOS) database on 27 March, 2020. This database is one of the most popular sources among researchers and academics and has been specifically designed for evaluating importance and influence of scientific publications through journal impact factor, despite this system being an ongoing matter of debate. Moreover, WOS offers a wide content coverage and provides very detailed information for citation analysis [14]. The search was conducted using the terms “telomere length” and “child” by the topic field that includes title, abstract, author keywords, and keyword plus terms. Another search was also performed in parallel using only the term “telomere length” to compare the retrieved information obtained from the two searches. All references indexed and published until 31 December, 2019 were included for the analysis. When obtaining the raw data of the bibliographic search, we carried out a manual revision of the documents found. To ensure the accuracy of the search and to minimize any mistakes of the data provided by the database, two researchers (D.P.-B. and E.-M.N.-M.) independently examined all the retrieved documents in order to identify publications that were not related to the field of our study, or missing information in the data we wished to analyze. The following data were extracted from each publication: title, journal, article type, author names and affiliations, keywords, date of publication, research area, and abstract. 

### 2.2. Data Analysis and Visualization

Based on the data exported from the WOS database and then completed by the team researchers (D.P.-B. and E.-M.N.-M.), the bibliometric analysis was conducted using R software version 3.6.2 (R Foundation for Statistical Computing, Vienna, Austria; http://www.r-project.org) through the Bibliometrix R package [15]. Based on the annual scientific production, this package provides a quantitative analysis performing data matrices and using the main bibliometric methods for a comprehensive science mapping analysis. Scientific production and collaboration were calculated and ranked based on authors, journal sources, countries and institutions, the most cited papers, and the most used terms. The type of documents, general categories and subject areas were classified using the intrinsic function of the WOS. The influence and quality of journals were also measured using the impact factor obtained from the latest Journal Citation Reports (2018) elaborated by Clarivate Analytics. In addition, GunnMap 2 (http://lert.co.nz/map/) was used to create a world map to display geographical distribution of publications on telomere length in children.

## 3. Results

### 3.1. Publication Analysis Based on Numbers

**Figure 1:**
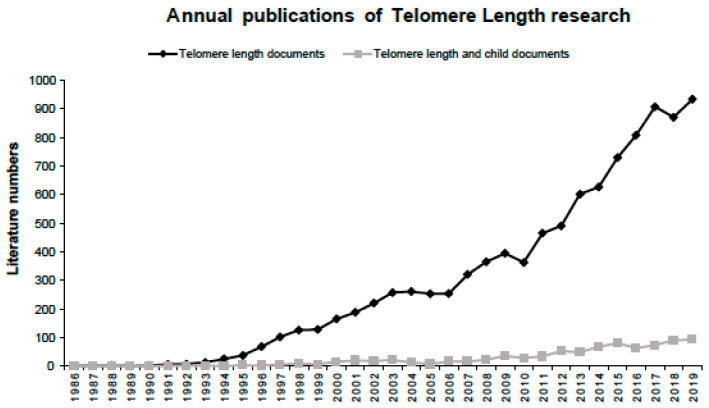
Annual distribution of publications on all telomere length research and telomere length in children research.

The search strategy yielded a total of 840 publications on telomere length in children. It represented 8.4% of the whole of the retrieved documents with respect to all telomere length research (*n* = 9998). The annual evolution of the scientific production on all telomere length research and on telomere length in children research is displayed in Figure 1. The first article published on telomere length in children was dated 1991 and the annual growth rate in this research area was of 17.3%. Although the number of publications was moderate–low from its inception to 2008, the production increased considerably from 2012 and it has improved gradually since then. The highest number of publications was produced in 2019, totaling 92 documents. In contrast, the annual publication in all telomere length research was exponentially rising since 1993 (annual growth rate of 23%), reaching its maximum peak in 2019 with 934 documents. 

**Figure 2:**
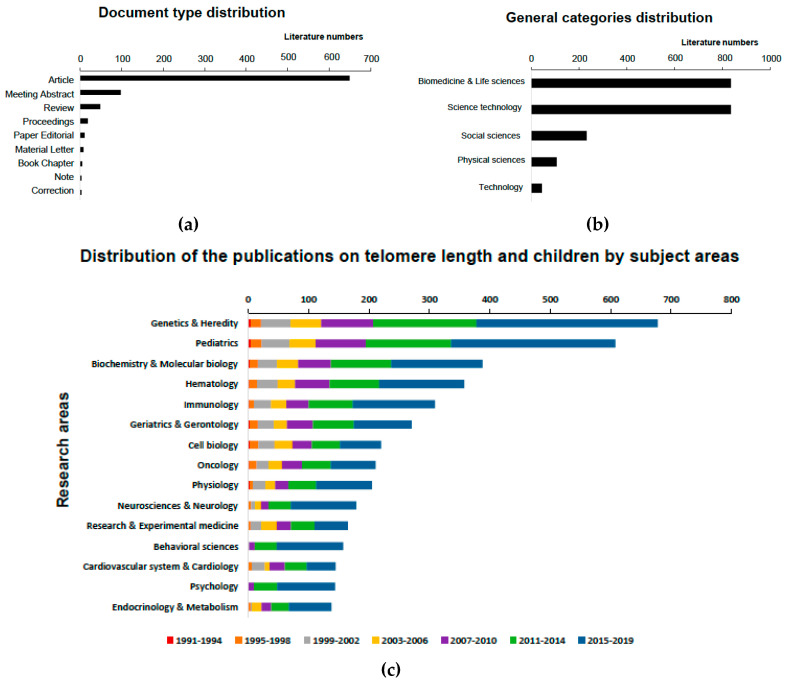
Distribution of the publications on telomere length in children by document type (**a**), general categories (**b**), and subject areas (**c**) over the period from 1991 to 2019.

Figure 2 shows the general characteristics about the distribution of the publications on telomere length in children by document type (a), general categories (b), and subject areas (c) over the period from 1991 to 2019. The majority of the retrieved publications were research articles (*n* = 649, 77.3%). Other documents were meeting abstracts (*n* = 98, 11.7%), reviews (*n* = 48, 5.7%), and proceedings papers (*n* = 18, 2.2%). To a much lesser extent (*n* = 27, ≤1%) editorial material, letters, book chapters, notes, and corrections were also published (Figure 2a). According to the general categories (Figure 2b), the publications were mainly classified into both Biomedicine and life sciences and Science technology (*n* = 836, 99.5%), although a notable number of references were published in Social sciences (*n* = 232, 27.6%) and Physical sciences (*n* = 107, 12.7%). Regarding the most frequently studied subject areas (Figure 2c), a significant amount of research in this field has been published in Genetics and Heredity (*n* = 678), Pediatrics (*n* = 608), Biochemistry and Molecular biology (*n* = 388), and Hematology (*n* = 358) from 1991 to 2019. During the most recent two periods, i.e., 2011–2014 and 2015–2019, these subject areas had also the highest production, although a substantial increase in research on Neurosciences and Neurology (*n* = 145), Behavioral sciences (*n* = 146), and Psychology (*n* = 134) was also evident and almost entirely published in the latest period 2015–2019. 

### 3.2. Publication Analysis Based on Journals

All the retrieved 840 documents were based on 407 different sources. Since the most frequent document type was research article (Figure 2a), it was assumed that journals were the most prominent sources of the publications. A total of 275 (67.6%) sources published at least one document, 106 (26%) published between two and four, 18 (4.4%) between five and nine, and eight (2%) indexed 10 or more references.

Table 1 discloses general information about the top 20 most prolific journals publishing articles on telomere length and children. A total of 252 references were listed among the top 20 most prolific journals accounting for 30% of all the publications on telomere length in children. The highest scientific production on this research area was compiled from high ranked journals primarily focused on hematology, oncology, geriatrics and gerontology, and multidisciplinary sciences. Blood (*n* = 65) and PloS One (*n* = 34) were the journals that published 39.3% of the references, which represented 11.7% of the total publication on this subject (*n* = 840). 

### 3.3. Publication Analysis Based on Authors

A total of 4804 authors participated in the publication of all 840 references included, with a mean of 5.7 authors per document and of 0.2 documents per author. There were 23 authors of single-authored documents and 4781 authors of multi-authored documents. Given that the number of occasions that an author appeared corresponded with the number of documents published by each one, there were 6839 author appearances. The number of authors who had a single document, i.e., transience index, was 3762, which represented 78.3% of all the authors. Among the rest of the authors who published more than one article, there were 934 who published between two and four documents, 84 between five and nine, and only 24 had 10 or more references (0.5%) in this research area. The authors who had the higher number of publications (≥20) were Savage, S.A. (*n* = 27), Lin J. (*n* = 26), Lansdorp, P.M. (*n* = 22) and Alter, B.P. (*n* = 20). Co-authorship analysis indicated a mean of 8.14 co-authors per document and a collaboration index of 5.86, i.e., total authors of multi-authored documents (*n* = 4781) divided by total multi-authored documents (*n* = 816). 

### 3.4. Publication Analysis Based on Countries/Regions and Institutions

**Figure 3:**
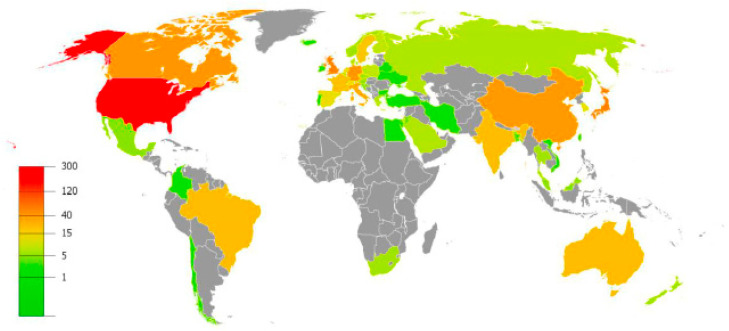
Geographical distribution map of the publications on telomere length in children.

The information on countries/regions and institutions was obtained from the first author’s country affiliation. The retrieved publications were geographically distributed through 53 countries over five continents (Figure 3). There were 15 countries that only published one article, followed by 20 countries that published from two up to nine articles, and 16 countries contributed more than 10 articles, accounting for 29.4%, 39.2%, and 31.4% of the total publication productivity, respectively. Table 2 displays general features of the top 20 most prolific countries in publishing on telomere length in children. The most productive country was USA that concentrated over a third of the scientific production (*n* = 300, 35.7%), followed by Japan and United Kingdom (*n* = 59, 7.0%), Canada and Germany (*n* = 43, 5.1%), and China (*n* = 41, 4.9%). In terms of intra-country collaboration, USA presented the highest number of articles published by authors from the same country (*n* = 206), ensued by Japan (*n* = 49), United Kingdom (*n* = 39), China (*n* = 38), Canada (*n* = 28), and Italy (*n* = 25). Regarding inter-country collaboration, the countries with higher number of publications with authors from different countries were USA (*n* = 94), Germany (*n* = 25), United Kingdom (*n* = 20), and Canada (*n* = 15), although in relative terms, the highest values were for Belgium and Finland (multiple country publication (MCP) ratio = 0.7, total documents = 6), followed by Germany and Netherlands (MCP ratio = 0.6, total documents = 43 and 15, respectively). 

**Figure 4:**
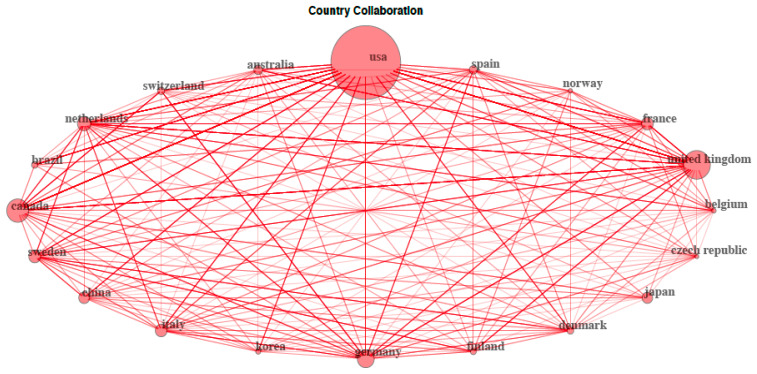
Country collaboration network map. Each link shows a collaboration between two countries and larger circle size indicates a higher number of international collaborative projects conducted by each country.

Figure 4 shows the analysis of the international collaboration in the top 20 most collaborative countries. As observed, every link displays a collaborative relationship between two countries and the circle size indicates the amount of collaborations established by each country. Among the top 20 most collaborative countries, the number of bilateral relationships created between countries was generally similar (*n* ≥ 15), except for Switzerland (*n* = 14), Norway (*n* = 12), Japan (*n* = 11), and Brazil (*n* = 8). However, the total number of international collaborative projects conducted by each country showed relevant differences. The country that established more international collaborations was USA (*n* = 223), followed by United Kingdom (*n* = 138), Canada (*n* = 119), Netherlands (*n* = 105), and Germany (*n* = 103). Table 3 displays general characteristics of the top 20 most prolific research institutions in publishing on telomere length in children. Of all 840 retrieved documents these institutions published 183 references (21.8%), of which 114 (62.3%) were from institutions located in USA. Along with Canadian (*n* = 18) and Brazilian (*n* = 10) institutions, 78.1% of the research publication was produced in research centers situated in the Americas, accounting for 17% of the total production. The rest of the research findings were published from institutions in Japan (*n* = 21, 11.5%) and Europe (*n* = 19, 10.4%), both accounting for 5% of total published references.

### 3.5. Publication Analysis Based on Citations

The impact of publications or authors that contributed to the available research on telomere length in children was based on a total of 28,226 citations from all 840 retrieved documents, with a mean of 33.6 citations per document. There were 154 (18.3%) documents with no citations, 53 (6.3%) with at least one citation, 208 (24.8%) that have been cited from two to nine times, 277 (33%) from 10 to 49, 79 (9.4%) from 50 to 99, and 65 (8.2%) that have received at least 100 citations. Since the first published article, the mean number of citations per year was 3.4, although considering different periods of time, the mean number per year was 6.4, 3.4, and 3.3 for the periods 1991–1999, 2000–2009, 2010–2019, respectively. A total of 272,220 citations were given by all the authors (*n* = 4804) from the 840 retrieved documents, with a mean number of citations per author of 56.7. There were 547 (11.4%) authors with no citations, 258 (5.4%) with at least one citation, 1156 (24.1%) that have received from two to nine, 1660 (34.5%) from 10 to 49, 421 (8.8%) from 50 to 99, and 761 (15.8%) that have been cited at least 100 times. In terms of countries, the highest number of citations was observed in USA (*n* = 11,260), United Kingdom (*n* = 3119), Japan (*n* = 2335), Canada (*n* = 1967), and Germany (*n* = 1772), with an average article citations of 41.3%, 58.9%, 41.7%, 45.7%, and 45.4%, respectively.

As shown in Table 4, among the top 20 most cited authors, five have received ≥1000 citations, six have been cited ≥500 times, and only two have not still been reached 100 citations. Overall, those authors who had the largest number of citations also had the highest h-index (i.e., h-index > 10). 

However, Blackburn E.H. who has been cited a total of 943 times had a h-index of 7 while Takubo, K. with 576 citations had a h-index of 11. Moreover, a bigger number of publications was an indicative of a higher author’s scientific productivity as measured by the h-index and/or the g-index, except for Kojima, S. (h-index = 4, g-index = 9) who, having 16 publications, has only received 94 citations so far. Considering the length of academic career, the m-index showed that Savage, S.A. (0.9), Lin, J.(1.3), Epel, E.S. (0.9), Giri, N. (0.8), Drury, S.S. (1.0), Blackburn, E.H (0.7), and Li, Y. (0.7) were the authors who had higher growth in his/her scientific productivity. 

The top 20 most cited research papers on telomere length in children are listed in Table 5. A total of 19 different scientific journals published the top 20 most cited papers, of which two were published in Blood (Alter, B.P. et al., 2009, 2007). These journals were high-impact journals (15 ranked in the 1st Journal Citation Reports (JCR ) quartile) and mainly focused on publishing research on oncology (two journals), immunology (one journal), genetics (four journals), cell biology (three journals), multidisciplinary sciences (one journal), experimental medicine (one journal), hematology (two journals), psychiatry (two journals), public health (one journal), general medicine (two journals), and geriatrics (one journal). Until now, the more highly cited papers on telomere length in children have been cited >200 times. The most cited research paper entitled “Adoptive Cell Therapy for Patients with Metastatic Melanoma: Evaluation of Intensive Myeloablative Chemoradiation Preparative Regimens” was published in the Journal of Clinical Oncology (Impact Factor (2018): 28.245) in 2008 and has received 863 citations, with a mean number of 66.3 citations per year. Among the authors contributing the top 20 most cited papers, there were some of the most cited authors (Table 4): Landsdorp, P.M. (three papers), Blackburn, E.H. (one paper), Takubo, K. (one paper), Alter, B.P. (three papers), Giri, N. (two papers), and Savage, A.S. (two papers). 

### 3.6. Publication Analysis Based on Terms Frequency

**Figure 5:**
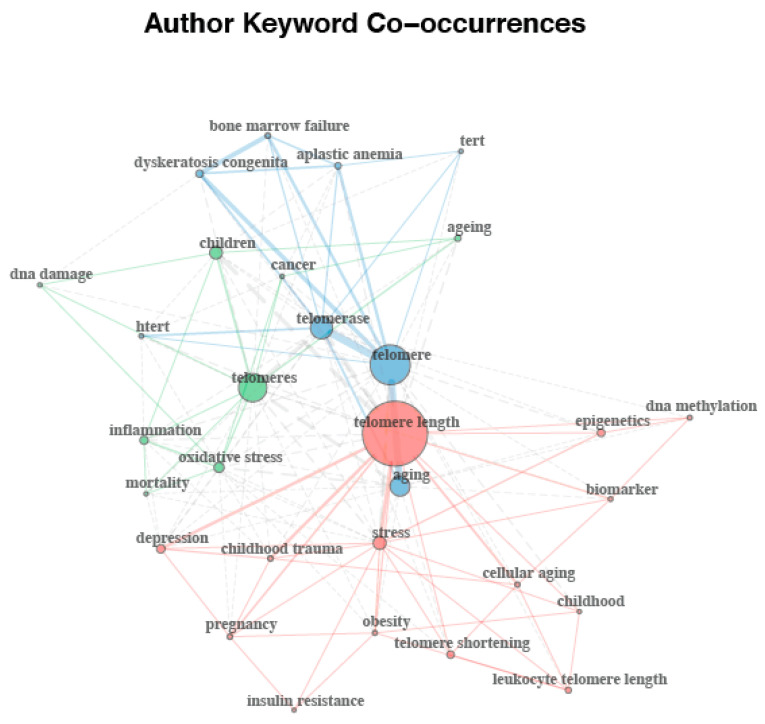
Author keyword co-occurrences network map.

**Figure 6:**
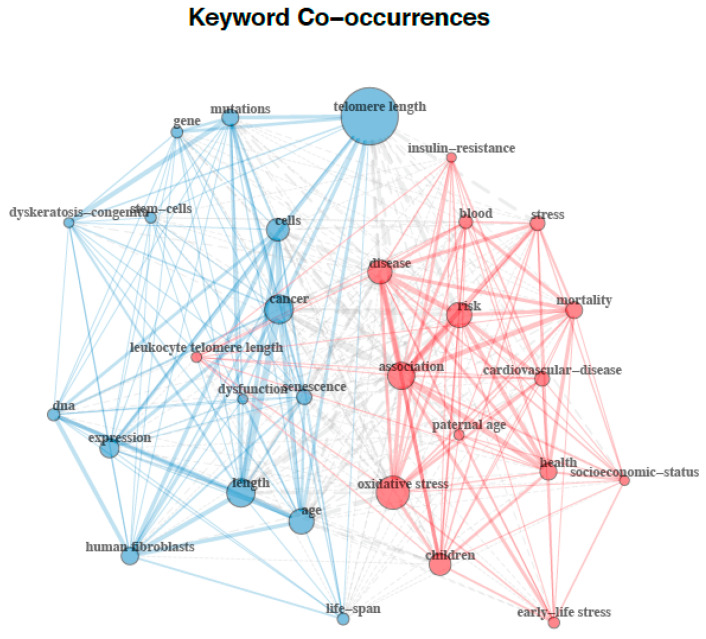
Keyword plus terms co-occurrences network map.

The analyses of the 30 most frequently used terms included in all the retrieved documents using the author keywords and the keyword plus terms (i.e., keywords associated to the manuscript by Thomson Reuters’ Institute for Scientific Information (ISI) WOS databases) are displayed in Figure 5 and Figure 6, respectively. A total of 1403 author keywords were retrieved, appearing 2503 times overall. The 30 most frequently used author keywords occurred from a minimum of seven to a maximum of 667 times. Based on the higher frequency of keywords and the proximity of their connections, as reflected by the larger size of nodes and the thickness of lines respectively, network analysis of the author keyword co-occurrences revealed a structure of links between keywords divided into three different clusters (Figure 5). The author keywords or terms with the highest occurrence were telomere length (*n* = 138), telomere (*n* = 97), telomeres (*n* = 56), telomerase (*n* = 49), aging (*n* = 43), children (*n* = 28), and stress (*n* = 22). A total of 2176 keyword plus terms were encountered and showed a maximum of 6301 times. The 30 most frequently used keyword plus terms were found from a minimum of 24 to a maximum of 1681 times. As shown in Figure 6, two clusters resulting from the network analysis of the keyword co-occurrences were identified as knowledge structure of research on telomere length in children. The keyword plus terms used with the highest frequency were telomere length (*n* = 156), cancer (*n* = 102), association (*n* = 100), length (*n* = 95), oxidative stress (*n* = 93), age (*n* = 87), risk (*n* = 85), disease (*n* = 83), cells (*n* = 78), and children (*n* = 65).

## 4. Discussion

The present bibliometric study shows for the first time a comprehensive analysis of the academic research on telomere length in children. The analysis of the scientific publication evolution on telomere length from its inception to 2019 revealed that investigation into this issue in early life has gained importance during the last decade, thus becoming a relevant field of research. Since 2012, the number of publications on telomere length in children has notably increased and its overall trend reflects a steady improvement. A large body of the research in this field has been published in journals dedicated to biomedical and biotechnological sciences, or more specifically, in journals mainly covering findings from genetics, pediatrics, cell biology, and hematology research. However, in recent years, neuroscience and behavioral sciences have also emerged as especially active areas of scientific interest.

Compared to all telomere length research, the number of publications on telomere length in children was considerably lower. One of the most likely explanations may be that telomere length is widely known as a biomarker of biological aging [2], and consequently, it has mainly been examined within the context of human aging-related diseases [1]. This point may also help to clarify that geriatrics and gerontology have been seen as important areas of research intrinsically linked to the study of telomere length measured at early ages due to, in all likelihood, its potential causal role connecting early adversity and later disease. Moreover, although the first investigations date back to 1991, it should be noted that the interest in studying telomere length at early stage of life is relatively recent, since a large body of research has been mainly conducted in the last decade. Nevertheless, the evolution of publication in this field shows a continued growth from the start in line with that observed in all telomere length research.

Notwithstanding the increment in research output in the most recent years, this study shows that a considerable amount of the publication on telomere length in children could in part come from studies presenting preliminary results. Among other likely reasons, this could be suggested by the fact that there was a high transience index, according to which nearly 80% of the authors had only published a single article. Moreover, based on the distribution of publications by research areas, it was observed that the development of several existing research areas has been very gradual and that there have been rapid advances in newly emerging areas of study during the recent years. In this regard, considering the annual publication numbers, this seems to indicate that there is a considerable variety of approaches for the study of telomere length in children and that most of these approaches are still at an early stage of development. In fact, the results regarding the most prolific journals showed that research findings are published in journals focused on different subject areas such hematology, multidisciplinary sciences, psychiatry, oncology, or cell biology among others, which verifies the broad scope of this research field. As indicated recently, a transdisciplinary approach to understanding the active role of telomere is a highly distinctive feature of research on telomere length in children [7,8,11,12]. In this sense, it might be argued that the case that this research field is developing into a variety of subject areas could be seen as an indicator of low-quality research publication. However, an intriguing finding of our study was that these journals are highly ranked journals and that they published around a third of indexed research on telomere length in children, suggesting that an important body of research attains the standards of scientific soundness. Thus, although the publication numbers were found to be moderate, an important amount of scientific output is from highest-quality journals.

Geographical distribution of publication on telomere length in children was mainly located in North America (USA and Canada), followed by Europe (United Kingdom and Germany) and Asia (Japan and China). The USA is by far the world’s leading country in scientific production in this field. This includes the highest number of research articles, international collaborations and research institutions. In fact, part of the production of some of the most prolific countries is, to a large extent, a direct result of the international collaboration with USA. These results are consistent with that observed in a recent review about publication trends from top publishing countries [16]. This study also showed that the USA is the world’s leading exponent of research publications. Similarly, when analyzing the country collaboration network, although USA maintains a dominant position, Europe along with other countries seem to exert an evident counterbalance to the mapping of research [16]. In this sense, apart from some known American institutions, it should be mentioned that other highly prestigious research centers situated in Japan, Brazil, Canada, and certain European countries are also important contributors to scientific production in this research on telomere length in children. 

Genetic studies, compared to other areas of research, have a limited number of publications describing their clinical significance [17]. In this regard, it is recognized that there are gaps that prevent the clinical translation of scientific findings about genetic markers [18] due to, in part, the lack of epidemiological research, thus reducing their scientific impact in parallel. In fact, the role of research and clinical applications of genomics are currently controversial issues that have opened a lively debate over biomedical research data and a relatively novel approach known as precision medicine [19,20,21,22]. Thus, regarding telomere length in children it would be reasonably expect that, since it is an ancillary topic derived from the knowledge about the role of telomere length, its body of research is still lacking [12] and its scientific impact is also lower accordingly. However, despite this limitation, the analysis of citation data showed that there is a notable growing metrics of scientific impact about the telomere length’s association with childhood. Based on author’s metrics, it was observed that some of the most cited authors have had a rapid academic career, suggesting a higher scientific productivity in a short period of time. Indeed, this would be consistent with the pattern of growth in the number of publications seen in recent years since around 2012. Moreover, according to data on the most cited papers, most of research findings have been published in highly selective journals, which reflects, to a considerable extent, the scientific quality of articles [23]. 

Interestingly, the findings from the keyword co-occurrence network of this study offer an explicit snapshot of the current state of telomere length in children as a topic of research. By examining the links between keywords in literature, this type of analysis can disclose core concepts and knowledge structure of a scientific field [24]. Although less clear, author’s keyword network revealed that this research field is focused on three main issues: (1) those relating to telomere biology; (2) those relating telomeres and inflammation processes in children; and (3) those relating telomere length and its health effects. Keyword networks from the keyword plus terms displayed a paradigm split into two different areas of interest: (1) those relating to telomere length biology; and (2) those relating to epidemiological research. On balance of evidence, it can be suggested that there are two main lines of research on telomere length in children. One line of research seems to be focused on providing a thorough understanding of the biological processes involved in telomere length and the other one seems to on identifying risk factors of diseases associated with telomere length in children.

Nevertheless, this study presents several potential limitations that should be acknowledged. We used the WOS database for searching and retrieving bibliographic data that were subsequently imported using Bibliometrix R package to conduct the analysis. Although this database is one of the most popular sources among researchers and academics to assess the importance and influence of scientific publications, it might have several disadvantages [14,25]. WOS offers a wide content coverage with very detailed information for citation analysis, although Scopus provides a more comprehensive overview of the world’s research output [14]. Moreover, Scopus can give, to a certain degree, more accurate information about author names because authors are always matched to their affiliations [14]. Nevertheless, all the bibliographic information was independently revised by two researchers (D.P.-B. and E.-M.N.-M.) in order to detect misleading and/or missing data that were then corrected and/or completed, where necessary. While considering these limitations, we believe that the findings of this study show relevant insights into the scientific production in telomere length in children, thus representing a valuable resource for scientific researchers and clinicians in this field. In this regard, we should emphasize that the bibliometric analysis performed using Bibliometrix has been also used by other authors and, in some way, our results may be comparable with others in the future.

## 5. Conclusions

This study shows that telomere length in children is a topic of research that has gained significant relevance in the last decade. The evolution of scientific production in this field was gradual although a notable increase in the number of publications was observed in recent years, and particularly in certain study areas. Currently, research on telomere length in children is formed of a wide range of different study areas although, in all likelihood, there seem to be notable efforts targeted at developing two main lines of research. One line of research is primarily focused on seeking a better understanding of the biological processes involved in telomere length including a detailed knowledge of both structure and function of telomeres, and the other one is based on an epidemiological approach that seeks to gain a proper understanding of how disease processes are related to telomere length during childhood. The present study can be used as bibliometric pattern for scientific researchers and clinicians in this field. In this regard, we hope that this study can help to find research hot spots and gaps by providing comprehensive analyses and structured information on this topic.

## Figures and Tables

**Table 1:** Top 20 most prolific journals publishing papers on telomere length in children.

Rank	Journals	Number of Articles (%) ^a^	Categories (Rank)	IF (JCR) ^b^
1st	Blood	65 (7.7)	Hematology (1/13)	16.601
2nd	PLoS One	34 (4.0)	Multidisciplinary Sciences (24/69)	2.776
3rd	Psychoneuroendocrinology	17 (2.0)	Endocrinology and Metabolism; Psychiatry; Neurosciences (36/145;36/146;76/267)	4.013
4th	British Journal of Haematology	12 (1.4)	Hematology (13/73)	5.206
5th	Experimental Gerontology	11 (1.3)	Geriatrics and Gerontology (24/53)	3.080
6th	Experimental Hematology	10 (1.2)	Medicine, Research and Experimental; Hematology (74/136;44/73)	2.462
6th	Hematological Oncology	10 (1.2)	Oncology; Hematology (92/230;24/73)	3.439
6th	Proc. Natl. Acad. Sci. USA	10 (1.2)	Multidisciplinary Sciences (7/69)	9.580
9th	Biological Psychiatry	9 (1.1)	Psychiatry; Neurosciences (7/146;12/267)	11.501
9th	Bone Marrow Transplantation	9 (1.1)	Immunology; Transplantation; Oncology; Hematology (46/158;4/25;60/230;16/73)	4.674
9th	Scientific Reports	9 (1.1)	Multidisciplinary Sciences (15/69)	4.011
12th	Mechanisms of Ageing and Development	8 (1.0)	Cell Biology; Geriatrics and Gerontology (91/193;18/53)	3.603
13th	Pediatric Blood & Cancer	7 (0.8)	Oncology; Hematology; Pediatrics (151/230;43/73;29/125)	2.486
14th	Aging Cell	6 (0.7)	Cell Biology, Geriatrics and Gerontology (31/193;3/53)	7.346
14th	American Journal of Human Biology	6 (0.7)	Biology (55/87)	1.438
14th	Cancer Research	6 (0.7)	Oncology (21/230)	8.378
14th	Development and Psychopathology	6 (0.7)	Psychology, Developmental (14/76)	3.593
14th	Journal of Affective Disorders	6 (0.7)	Clinical Neurology; Psychiatry (44/199; 32/146)	4.084
14th	Leukemia	6 (0.7)	Oncology; Hematology (14/230;4/73)	9.944
20th	American Journal of Human Genetics	5 (0.6)	Genetics and Heredity (9/174)	9.924

Abbreviations: IF (JCR), impact factor (Journal Citations Reports); Proc. Natl. Acad. Sci. USA, Proceedings of the National Academy of Sciences of the USA. ^a^ Percentage calculated out of the retrieved 840 documents. ^b^ Impact factor obtained from the Journal Citation Reports (2018).

**Table 2:** Top 20 most prolific countries in publishing papers on telomere length in children.

Countries	Total Number of Documents	% ^a^	SCP	MCP	MCP Ratio ^b^
USA	300	35.7	206	94	0.3
Japan	59	7.0	49	10	0.2
United Kingdom	59	7.0	39	20	0.3
Canada	43	5.1	28	15	0.3
Germany	43	5.1	18	25	0.6
China	41	4.9	38	3	0.1
Italy	33	3.9	25	8	0.2
Australia	23	2.7	14	9	0.4
Brazil	23	2.7	13	10	0.4
India	22	2.6	19	3	0.1
France	21	2.5	11	10	0.5
Sweden	19	2.3	12	7	0.4
Netherlands	15	1.8	6	9	0.6
Switzerland	13	1.5	6	7	0.5
Spain	12	1.4	9	3	0.3
Korea	11	1.3	7	4	0.4
Israel	9	1.1	6	3	0.3
Belgium	6	0.7	2	4	0.7
Finland	6	0.7	2	4	0.7
Poland	6	0.7	5	1	0.2

Abbreviations: SCP, single country publications; MCP, multiple country publications. ^a^ Percentage calculated out of the retrieved 840 documents. ^b^ Multiple country publication ratio was calculated as MCP divided by the total of published documents per country.

**Table 3:** Top 20 most prolific research institutes in publishing on telomere length in children sorted by the total number of articles.

Research Institute	Country	Number of Articles	% ^a^
Clinical Genetics Branch (NCI division)	USA	21	2.5
University of California San Francisco	USA	20	2.4
Tulane University	USA	16	1.9
Nagoya University	Japan	13	1.5
University of Sao Paulo	Brazil	11	1.3
University of Washington	USA	10	1.2
Duke University	USA	8	1.0
Tokyo Metropolitan Institute of Gerontology	Japan	8	1.0
Baylor College of Medicine	USA	7	0.8
Butler Hospital	USA	7	0.8
Columbia University	USA	7	0.8
Umea University	Sweden	7	0.8
University of Medicine and Dentistry of New Jersey	USA	7	0.8
University of British Columbia	Canada	6	0.7
University of California Los Angeles	USA	6	0.7
Queen Mary University of London	UK	6	0.7
Terry Fox Laboratory	Canada	6	0.7
Murdoch Children’s Research Institute	Canada	6	0.7
Zurich University	Germany	6	0.7
Harvard University	USA	5	0.6

Abbreviations: NCI, National Cancer Institute. ^a^ Percentage calculated out of the retrieved 840 documents.

**Table 4:** Top 20 most cited authors publishing on telomere length in children sorted by number of citations.

Author	H-index	G-index	M-index	TC	NP	YFP
Savage, S.A.	13	27	0.9	1120	27	2006
Lin, J.	13	26	1.3	1044	26	2011
Lansdorp, P.M.	16	22	0.7	1791	22	1999
Alter, B.P.	12	20	0.6	1051	20	2002
Giri, N.	11	18	0.8	1007	18	2007
Aviv, A.	12	17	0.6	837	17	2000
Calado, R.T.	9	17	0.6	373	17	2006
Epel, E.S.	11	17	0.9	792	17	2009
Young, N.S.	9	17	0.4	576	17	2001
Kojima, S.	4	9	0.3	94	16	2006
Kimura, M.	11	14	0.5	975	14	2000
Takubo, K.	11	14	0.5	576	14	2000
Drury, S.S.	9	13	1	454	13	2012
Baerlocher, G.M.	5	11	0.2	461	11	2001
Blacburn, E.H.	7	11	0.7	943	11	2011
Gadalla, S.M.	6	11	0.5	186	11	2010
Hama, A.	3	9	0.2	83	11	2006
Vulliamy, T.	7	11	0.4	318	11	2003
Arai, T.	8	10	0.4	487	10	2000
Li, Y.	4	7	0.7	160	7	2015

Abbreviations: TC, total citations; NP, number of publications; YFP, year of first indexed publication.

**Table 5:** Top 20 most cited research papers on telomere length in children.

Ranking	Author/s	Title	Year	Journal	TC	TC_Y_	IF
1st	Dudley, M.E. et al.	Adoptive Cell Therapy for Patients with Metastatic Melanoma: Evaluation of Intensive Myeloablative Chemoradiation Preparative Regimens	2008	JCO	863	66.3	28.349
2nd	Hiyama, K. et al.	Activation of Telomerase in Human Lymphocytes and Hematopoietic Progenitor Cells	1995	Journal of Immunology	791	30.4	4.718
3rd	Slagboom, P.E. et al.	Genetic determination of telomere size in humans: a twin study of three age groups	1994	AJHG	569	21.1	9.924
4th	Bonab, M.M. et al.	Aging of Mesenchymal Stem Cell in Vitro	2006	BMC Cell Biology	489	32.6	3.485
5th	Chang, E. & Harley, C.B.	Telomere Length and Replicative Aging in Human Vascular Tissues	1995	PNAS	484	18.6	9.580
6th	Rufer, N. et al.	Telomere Fluorescence Measurements in Granulocytes and T Lymphocyte Subsets Point to a High Turnover of Hematopoietic Stem Cells and Memory T Cells in Early Childhood	1999	Journal of Experimental Medicine	468	21.3	10.892
7th	Armanios, M. & Blackburn, E.H.	The telomere syndromes	2012	Nature Reviews Genetics	460	51.1	43.704
8th	Rausch, T. et al.	Genome Sequencing of Pediatric Medulloblastoma Links Catastrophic DNA Rearrangements with TP53 Mutations	2012	Cell	436	48.4	36.216
9th	Lindsey, J. et al.	In Vivo Loss of Telomeric Repeats with Age in Humans	1991	Mutation Research	380	12.7	2.107
10th	Marioni, R.E. et al.	DNA Methylation Age of Blood Predicts All-Cause Mortality in Later Life	2015	Genome Biology	372	62	14.028
11th	Wiemann, S.U. et al.	Hepatocyte telomere shortening and senescence are general markers of human liver cirrhosis	2002	FASEB Journal	309	16.3	5.391
12th	Alter, B.P. et al.	Cancer in Dyskeratosis Congenita	2009	Blood	241	20.1	16.601
13th	Shalev, I. et al.	Exposure to Violence During Childhood Is Associated with Telomere Erosion From 5 to 10 Years of Age: A Longitudinal Study	2013	Molecular Psychiatry	233	29.1	11.973
14th	Kiecot-Glaser, J.K. et al.	Childhood Adversity Heightens the Impact of Later-Life Caregiving Stress on Telomere Length and Inflammation	2011	Psychosomatic Medicine	232	23.2	3.937
15th	Deary, I.J. et al.	Cohort Profile: The Lothian Birth Cohorts of 1921 and 1936	2012	IJE	218	24.2	7.339
16th	Langford, L.A. et al.	Telomerase activity in human brain tumours	1995	Lancet	213	8.2	59.102
17th	Takubo, K. et al.	Telomere Lengths Are Characteristic in Each Human Individual	2002	Experimental Gerontology	210	11	3.080
18th	Cheung, N.K.V. et al.	Association of Age at Diagnosis and Genetic Mutations in Patients with Neuroblastoma	2012	JAMA	206	22.9	51.273
19th	Villani, A. et al.	Biochemical and Imaging Surveillance in Germline TP53 Mutation Carriers with Li-Fraumeni Syndrome: A Prospective Observational Study	2011	Lancet Oncology	205	20.5	35.386
20th	Alter, B.P. et al.	Very Short Telomere Length by Flow Fluorescence in Situ Hybridization Identifies Patients With Dyskeratosis Congenita	2007	Blood	204	14.6	16.601

Abbreviations: TC, total citations; TC_Y_, total citations per year; IF, impact factor (Journal Citations Report 2018; JCO, Journal of Clinical Oncology; AJHG, American Journal of Human Genetics; PNAS, Proceedings of the National Academy of Sciences of the United States of America; IJE, International Journal of Epidemiology; JAMA, Journal of the American Medical Association.
